# Risk factors for squamous cell skin cancer in HIV

**DOI:** 10.1186/1750-9378-7-S1-O26

**Published:** 2012-04-19

**Authors:** Shehnaz K Hussain, Po-Yin Chang, Otoniel Martínez-Maza, Zuo-Feng Zhang, Lisa Jacobson, Roger Detels

**Affiliations:** 1 Department of Epidemiology, University of California, Los Angeles, CA, USA; 2 Jonsson Comprehensive Cancer Center, University of California, Los Angeles, CA, USA; 3 Department of Obstetrics and Gynecology, David Geffen School of Medicine at UCLA, Los Angeles, CA, USA; 4 Department of Microbiology, Immunology and Molecular Genetics, David Geffen School of Medicine at UCLA, Los Angeles, CA, USA; 5 UCLA AIDS Institute, Los Angeles, CA, USA; 6 Department of Epidemiology, Johns Hopkins University, Baltimore, MD, USA; 7 Division of Infectious Diseases, David Geffen School of Medicine at UCLA, Los Angeles, CA, USA

## Introduction

Squamous cell skin cancer (SCSC) risk is increased in the setting of HIV making it one of the most common non-AIDS defining cancers. However, very few studies have examined individual level risk factors for SCSC in the setting of HIV.

## Methods

The Multicenter AIDS Cohort Study, which includes 6,973 adult homosexual and bisexual men from four metropolitan areas in the U.S., was the study population used to address our aims. Incident SCSC diagnoses were initially captured through self-report on the biannual study visit questionnaire, and verified by review of pathology reports. Age-standardized incidence ratio was calculated to compare SCSC risk in HIV positive study participants to risk in HIV negatives. Multivariate Cox proportional hazards regression models were used to obtain hazard ratios (HR) and 95% confidence intervals (CIs) for the association between exposures of interest and SCSC risk. HIV positive participants entered the analysis on the date of their first HIV positive study visit and were followed until an SCSC diagnosis, death, or loss to follow-up.

## Results

We identified 55 pathologically confirmed SCSC cases, 39 in HIV positive participants and 16 in HIV negative participants. In the HIV positive population, all cases occurred among white, non-Hispanics, and 18 (46%) were HAART exposed prior to cancer diagnosis. HIV positive men were more than four times as likely as HIV negative men to be diagnosed with SCSC (SIR=4.64, 95% CI=3.15-6.83). In multivariate models including only white, non-Hispanic, HIV positive participants, SCSC risk increased with increasing age (HR=1.12, 95% CI=1.07-1.17 per year) and HIV load (HR=1.38, 95% CI=1.19-1.56 per log unit increase). SCSC risk decreased with increasing CD4+ T cell number (HR=0.45, 95% CI=0.34-0.59 per log unit). Adjustment for MACS study site and HAART exposure did not strongly influence these associations.

## Conclusions

Increasing age and HIV load, and decreasing CD4+T cell count, all suggesting decreased immunologic competence, were significantly associated with SCSC risk in the HIV-infected study population.

